# A systems-change approach to addressing the mortality surveillance gap in Pakistan

**DOI:** 10.7189/jogh.15.03027

**Published:** 2025-08-04

**Authors:** Mohummad Hassan Raza Raja, Zahra Hoodbhoy, Sana Sheikh, Muhammad Imran Nisar, Sajid Bashir Soofi, Sameen Siddiqi, Zafar Mirza, Faiza Bashir, Mirza Tayyab Mehmood, Zainab Samad

**Affiliations:** 1 Duke University, Department of Medicine, North Carolina, USA; 2 The Aga Khan University, Department of Pediatrics and Child Health, Karachi, Pakistan; 3 The Aga Khan University, Health Data Science Centre, Karachi, Pakistan; 4 The Aga Khan University, Department of Medicine, Karachi, Pakistan; 5 The Aga Khan University, Department of Community Health Sciences, Karachi, Pakistan; 6 Shifa Tameer-e-Millat University, Global Institute of Human Development, Islamabad, Pakistan; 7 National Institute of Health, National Bioethics Committee, Islamabad, Pakistan

## Abstract

With a lack of cause of death estimation and an inadequate and fragmented Civil Registration and Vital Statistics system, Pakistan faces a significant gap in data on mortality. This poses significant challenges for health policy planning and monitoring. In this viewpoint, we draw on systems-change frameworks to examine and provide recommendations to improve mortality surveillance in Pakistan. We use the multiple cause diagram framework to understand the challenges and barriers to instituting a robust mortality surveillance system in Pakistan. We also examine current and future scenarios and what it will take to get to best future scenarios using the Theory of Change model. Through the multiple cause diagram mapping, we show that the poorly functioning mortality surveillance system in Pakistan is underlain by multiple complex and interrelated multisectoral challenges. However, a cost-effective, agile, and data-lean system of mortality surveillance can exist through strengthening already existing systems. This could be accompanied with context- and resource-sensitive use of different types of surveillance methods such as verbal autopsy tools implemented in the community and integrated into sample registration systems, as well as hospital-based surveillance in urban areas with government coordination. This can be achieved with cross-sectoral, cross-agency collaboration, capacity strengthening, and local stakeholder involvement.

Pakistan is the 5th most populous nation in the world, with 64% of the population aged <30 years [1]. Due to its significant geostrategic importance, events and developments occurring in the country reverberate beyond its borders. From a global health perspective, Pakistan’s health outcomes remain of crucial importance, due to its young, rapidly growing population, and its growing diaspora. Improvement of health outcomes across age strata requires strategic policy planning based on accurate data. However, due to inadequate capture of vital statistics and lack of cause of death (COD) data, Pakistan risks formulating health policies that are not adapted to its context.

## CIVIL REGISTRATION AND VITAL STATISTICS SYSTEMS IN LOW- AND MIDDLE-INCOME COUNTRIES

One in two deaths go unreported globally, with most of the mortality data gaps lying in low-middle-income countries (LMICs) [2]. Civil registration and vital statistics (CRVS) systems record key data, such as births, deaths, migration, marriages, and divorces, within a country. They help in maintaining accurate demographic data and are the cornerstones for building an evidence base for health policy, planning, monitoring, and evaluation. Despite this, literature suggests that only one quarter of countries globally have a functioning CRVS system to support policy planning [3]. It is relatively easy to understand why this is the case: maintaining comprehensive CRVS systems in economically challenged nations with underdeveloped health care infrastructure is prohibitively costly on a national scale [4,5]. Historically, many high-income countries (HICs) took several decades to establish fully operational CRVS systems. Among other factors, this has generally depended on national priorities, as well as having adequate national resources and a trained physician workforce [6].

## EXISTING MORTALITY SURVEILLANCE SYSTEMS IN PAKISTAN

In Pakistan, a decentralised civil registration system is in place, wherein the responsibility for actual registration and legal obligations lies with local administrative bodies – specifically, in local union councils [7,8]. At that level, in theory, vital event information is digitally inputted into a Civil Registration Management System (CRMS) developed by the National Registration and Database Authority (NADRA), which has no authority over the registration process itself [7,8], but rather offers technical support for CRMS implementation and regularly reconciles vital event data with national databases. This reconciliation process involves periodic visits to each union council, where data is collected *via* USB devices and then transferred to the national population databases through NADRA district offices [9]. Despite this, there are shortfalls stemming from improper utilisation of the existing system due to inadequate training of union council staff; inadequate data capture due to a reliance on manual processes and lack of information technology infrastructure; and a lack of data flow stemming from the CRMS not being linked to any district level or provincial health information system, such as the District Health Information System 2 (DHIS2).

Currently, civil registration in Pakistan does not yield vital statistics due to its insufficient coverage, nor are current records used to process COD information [10]. Data from the World Health Organization (WHO) Survey, Count, Optimize, Review, Enable technical package estimates only 40% of births and 35% of deaths are registered in the country, with no data on the deaths registered with a medical certificate [11]. In fact, most vital statistics in Pakistan are derived and estimated from household surveys or censuses, which have several limitations in terms of their timeliness, frequency, completeness, reliability, and accuracy [9].

## FORMATIVE WORK IN DEVELOPING A CRVS SYSTEM IN PAKISTAN

The Government of Pakistan has previously carried out extensive groundwork, including the establishment of a National CRVS Steering Committee in 2014, with representatives from several ministries, development partners, and the Technical Support Unit [8]. From 2016 to 2020, several initiatives were undertaken to understand Pakistan’s current position, including an analysis of existing CRVS legislation, gap and stakeholder analysis, and evaluation of the CRVS operation [9,12,13]. While a national framework on CRVS reforms has been developed through formative work, this has not been implemented judiciously, as much of the existing policy work is yet to be translated into action. This has likely not yet occurred due to the immense work that needs to be done across sectors and domains, requiring significant financial investment, political stability, and continuity of governance. Thus, despite the extensive groundwork, a mortality data gap still exists.

## REASONS FOR A MORTALITY DATA GAP IN PAKISTAN

There are several interrelated, multisectoral barriers that exist for reporting death statistics in Pakistan, as displayed in our multiple cause diagram (Figure S1 in the **Online Supplementary Document**). Among many factors, these include inefficient health governance, perpetuated by inadequate political will and low health budget, leading to a fragmented and decompartmentalised health system, with individual entities acting siloed with different operating standards and expectations. An example of this is that the country’s DHIS 2 only registers information from public health facilities, despite most of the population choosing to seek care from private facilities [10,14,15]. Without legislation mandating for death certification, standard death certificate issuance (and consequently, reporting) is lacking and the practice of having unregulated graveyards/burials is predominant. Certain cultural and religious practices hinder death reporting, with a strong emphasis placed on carrying out last rites, such as washing the dead body and burying the deceased as soon as possible [16]. Both anecdotally and as noted in literature from other regions, these rituals are deeply rooted in religious beliefs and cultural norms, and often prioritise the spiritual and communal aspects of death over bureaucratic processes of death registration [17]. With 64% of the population living in rural parts of the country, deaths often occur in areas where formal medical care is lacking, and hence death registration does not take place [18]. In other cases, the lack of registration can simply be put down to lack of awareness of the importance of registration, especially among marginalised groups (transgender population, religious minorities, *etc*.) [9]. In other anecdotal instances, due to a potential loss of income from pensions or social support systems, families may not register the death of a family member, in order to continue to seek allotted benefits. There are also existing procedural barriers and accessibility barriers that hinder death reporting [9].

A lack of accurate data on these metrics is not only hampering progress towards the Sustainable Development Goals (SDGs) and universal health coverage (UHC), but also results in an inability to execute evidenced based, contextual, and strategic health policies [19]. The gap left by a largely non-functional CRVS system, leads to estimation of disease and death burdens *via* modelling and predictive instruments [20]. While improvements in data processing and modelling can lead to incremental improvements in the accuracy of estimates, fundamental improvements require efforts to improve primary data collection.

## A ROADMAP TO A CONTEXTUAL MORTALITY SURVEILLANCE SYSTEM

Nations with a similar level of socioeconomic development to Pakistan, such as Mozambique, Bangladesh, Vietnam, and Indonesia, have successfully implemented innovative and contextual methods for accurately capturing mortality statistics [21–24]. This would suggest cost-effective, agile, and data-lean mortality system can exist in Pakistan and can be achieved with cross-sectoral, cross-agency collaboration, capacity building, and local stakeholder involvement. Contextual use of different types of surveillance techniques such as verbal autopsy tools in the community integrated into sample registration systems and hospital death-based surveillance in urban areas with government coordination may be utilised based on each geographic unit’s existing resources and capacity.

**Figure 1:**
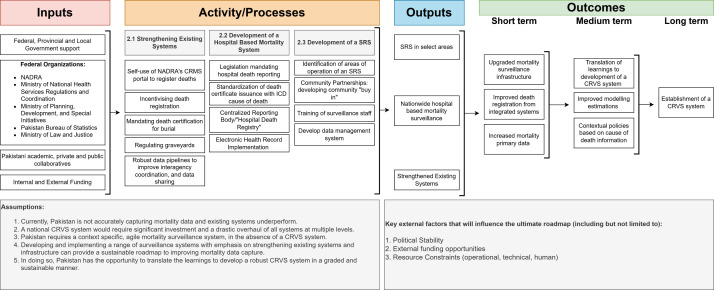
A roadmap to a contextual mortality surveillance system.

A Theory of Change model is a valuable framework for tracking progress toward specific goals, offering an outline of how and why a desired change is expected to occur [25]. It systematically maps out the essential steps needed to achieve long-term objectives, providing clarity on the path from actions to outcomes. *Via* one such model, we display the broad strokes of a roadmap that Pakistan could take to develop such an effective mortality surveillance system (**Figure 1**). This would include utilising a combination of approaches: strengthening existing systems, developing a hospital-based mortality surveillance system, and developing a sample registration system in select areas.

### Strengthening existing systems

Before developing new systems to capture mortality data, there is a need to strengthen existing systems in a sustainable, incremental manner. The opportunities to do so can be identified from the multiple cause diagram (Figure S1 in the **Online Supplementary Document**). This strategic approach not only maximises the utilisation of available resources, but will also cultivate a culture of continuous improvement, laying a solid foundation for the successful integration of new data capture systems in the future.

Priority rests on improving access and utilisation of death registration services. Currently registration of vital statistics (birth and deaths) occurs at designated NADRA centres. Taking advantage of the over 80% mobile teledensity, an online portal could be developed through which citizens would be able to register deaths on their own devices without having to travel to designated centres [25]. Increased access could ultimately lead to increased data registration. This, however, must be undertaken with adequate protections, such as e-signatures by witnesses, not only due to sensitive nature of the information, but also to enable factual reporting. Cross verification of information through documentation is also possible, in cases where ahe deceased’s family can provide standard death certificate signed by a health care professional. In cases where there is no standard death certificate, following registration, local health care workers and/or local government officials can visit the household where the death registration has occurred. Providing a value-added component to death registration, such as tying death registration to inheritance proceedings, may also help improve utilisation of death registration services. Additionally, there is a need for robust, yet actionable legislation such that is mandates all citizens to register deaths. Simultaneously, efforts to raise awareness about the significance of death registration should be actively pursued. Implementing targeted media campaigns and outreach initiatives can contribute significantly to educating the public and dispelling any existing misconceptions. The collective impact of these measures is instrumental in fostering a culture that recognises the importance of death registration, thereby contributing to increased utilisation of existing infrastructure for death registration.

To ensure all deaths are captured, graveyards and crematoriums would also need to be formally regulated. Urban areas are home to a patchwork of graveyards, ranging from those managed by local government authorities and non-governmental organisations to those that are independently run by mosques, religious organisations, and housing societies, including ceremonial graveyards. In rural areas, the deceased are likely to be buried in unregulated spaces, managed by local religious leaders or landowners. Regulation begins with establishing governance and creating legal frameworks that define the roles, responsibilities, and standards for graveyard management and monitoring. All graveyards and burial sites should be brought under the control of the local government, and each graveyard should have managing personnel that are able to record electronically the details of the deceased, with an appropriate standard death certificate being mandated before the burial is allowed to proceed. Ideally, a centralised database for graveyard mortality records should be developed. Monitoring teams should be established to oversee compliance with the regulatory frameworks by conducting regular inspections and ensuring that graveyards adhere to the defined standards.

Lack of a flow of data are also a key issue, whereby data are collected at times, but with an inadequate flow of information from the local to the provincial government, and (subsequently) to the federal government. Addressing this issue requires the establishment of robust data pipelines that facilitate the transfer of information from the grassroots to the federal level with appropriate coordination between relevant stakeholder agencies. Additionally, the systems need to be extensively digitised and automated. Data collection should ideally occur electronically, removing the need for paper-based inputs and data from each union council’s CRMS should be transmitted automatically to a centralised agency. The Ministry of National Health Services Regulations and Coordination should then be tasked with the aggregation and analysis of this data, ensuring it contributes to national health statistics and informs policy decisions.

Besides addressing the structural barriers described above and in the multiple cause diagram (Figure S1 in the **Online Supplementary Document**), digitisation of the current infrastructure with agile technologies and easy-to-use interfaces may also be one key to strengthening the current CRVS system and interagency data flow. According to the United Nations Children’s Fund (UNICEF), a fully-digitised CRVS system can significantly enhance coverage (including traditionally difficult-to-reach areas), standardise and simplify civil registration and vital statistics procedures, consolidate data from multiple systems, and securely store data at scale, all while remaining cost-effective [26]. While these benefits may seem worthwhile, digitising data and systems is a complex undertaking that requires years of strategic planning and must be introduced gradually. A well-thought-out long-term implementation roadmap is imperative. Iterative design and continuous stakeholder engagement would be key to implementing such a tool for CRVS systems.

### Development of a hospital-based mortality surveillance

The strengthening of existing systems to capture deaths from communities should be paralleled with an emphasis on collecting data from hospitals. Hospital-based deaths may represent a biased cohort of deaths; however, in urban areas with sufficient hospital coverage and use, they can provide valuable insights into health care trends, disease patterns, and overall public health outcomes. This approach will require an extensive overhaul of the current systems and procedures, in recognition of the areas of opportunities identified in the multiple cause diagram (Figure S1 in the **Online Supplementary Document**), with priorities areas resting on standardisation between public and private domains and digitisation of hospital information systems.

There needs to be standardisation across both private and public sectors regarding death certificate issuance regarding deaths within hospitals, which must be mandated by legislation to report deaths with appropriate documentation to either a centralised reporting body/registry or provincial health ministries (and subsequently the Ministry of National Health Services Regulations and Coordination) on a daily/weekly basis. The issued death certificate should be in standard format and contain an International Classification of Diseases (ICD) COD, appropriately signed off by the physician certifying the death. This will require training of physicians to appropriately assign cause of death as per standardised diagnoses codes. Additionally, the existing NADRA CRMS may potentially be integrated into all public and private health care facilities, with access provided for registration of hospital-based deaths with medical death certificates. The obligation to report deaths should be accompanied by stringent guidelines, ensuring that the reporting process is conducted in a non-punitive environment.

In parallel with these developments, there needs to a transition to digitised health systems. Hospitals across Pakistan employ a paper-based system, leading to challenges in not only collecting and storing data, but also transferring it across domains, levels, and sectors. While intermediate systems exist, such as the DHIS 2, they rely on data that is first collected in paper-based systems, after which it is compiled manually and inputted in the system. This process is inefficient and error prone. Furthermore, the DHIS 2 only gathers data from public sector facilities. A standard or interoperable electronic health record system across all public and private facilities would be required and would enable automatic transfer of data. As mentioned above, this transition will require significant investment in information technology (IT) infrastructure, training of personnel, and development of secure data transfer protocols to safeguard patient confidentiality and data integrity.

Together, these developments could lead to regular reporting of deaths from hospitals. Despite efforts to strengthen existing systems and establish a hospital-based mortality surveillance system, access to healthcare facilities remains suboptimal in numerous regions. Deaths in these underserved areas often occur at home, outside the purview of formal medical care, leading to underreporting and incomplete data. Therefore, even with the implementation of these measures, gaps in mortality data coverage are expected to persist. Addressing this issue requires innovative approaches tailored to the unique circumstances of these areas. As an example, one promising solution could be the establishment of a sample registration system that integrates an approach based on verbal autopsy (VA).

### Development of a sample registration system

#### An introduction to VAs

The term ‘Verbal Autopsy’ was first coined in India at the Narangwal project in the 1950s, although the practice of collecting information on death originates from pre-19th century Europe [27]. In essence, it denotes a practice of determining the COD based on verbal interviews carried out with the caregivers and/or the relatives of a deceased individual, in areas where medical autopsies or medical chart review for COD estimation is not routinely carried out. With over seventy years since they were first formally introduced, VA instruments have undergone significant evolution and standardisation to be contextually relevant. The main VA instruments in use have been developed by the WHO and, more recently, by the Population Health Metrics Research Consortium [26,28].

#### VA use in Pakistan

In general, VAs have predominantly been employed in research environments and as an integral component of extensive household surveys aimed at determining COD. Within Pakistan, they have been primarily conducted in funded small-scale community based or validation studies (Table S1 in the **Online Supplementary Document**). Among these studies, 16/18 (88.8%) have focussed on maternal and child health, highlighting the need to gather information regarding across all age strata. Yet, by being integrated in sample registration systems (SRSs), VAs have the potential to be employed as a surveillance technique in the country, as they have been in more than 45 LMICs for over 25 years [29].

#### SRSs in LMICs

An SRS involves gathering verbal autopsy data on deaths *via* community-based systems in several nationally representative clusters. This data can then be extrapolated to generate national statistics. Nowadays, SRSs are increasingly being used for this purpose in several LMICs, with the information leveraged *via* VAs being used to provide accurate statistics on COD distributions. Such systems, however, are only short- to medium-term solutions in regions where CRVS systems are not fully operational, acting as a type of a stepping stone [30]. An example of this is can be found in Mozambique, where the Countrywide Mortality Surveillance for Action platform launched in 2017. Under this project, 700 nationally representative clusters covering a population of 826 663 have been sampled annually since 2017 to generate national and subnational representative mortality rates and COD statistics, facilitating the enhancement of the country’s CRVS system [24].

Regionally in South Asia, the capacity of VAs to be integrated into national public health infrastructure and CRVS systems has additionally been explored by the Bloomberg Philanthropies Data for Health Initiative [28]. In Bangladesh, implementation of VA interviews in Kaliganj sub-district led to an increase in death registrations (within 45 days) from 458 to 1404 among a population of 304 600, giving the VA instrument >90% death registration coverage. This pilot was then scaled up by the Government of Bangladesh, with VAs implemented in a further 13 sub-districts covering a population of 4.8 million [23]. In Myanmar, through collaboration between the government and external funding partners, implementation of VAs was ultimately scaled up to 42 townships, covering a population of 8.2 million [29].

#### Steps to develop an SRS

Development of an SRS is a complex task that requires cross-sector and cross-disciplinary coordination. To ensure a successful rollout, various factors at both micro- and macro-levels must be considered, that are locally contextual. First, it is important to recognise that while national SRS systems have been established in other regions, this approach may not be financially viable for a country like Pakistan, which is both diverse and large in its geography. Mozambique with a population of just over 32 million, required a yearly investment of USD 1 million to implement a nationwide SRS; Pakistan, meanwhile, has a seven times larger population of over 247 million [31]. A nationwide SRS for Pakistan would be an expensive endeavour that would likely could not be sustainably transferred to local partners. Instead, in a fiscally conservative manner, SRSs should be primarily used in select areas on a sub-national basis, where there is a lack of hospital-based coverage or there is a marked inequity, such as to tabulate deaths in underrepresented communities, complementing existing systems.

Activities in developing SRSs include adapting the VA instrument to fit the local context, training staff to conduct mapping of clusters, surveys, and interviews, closely monitoring data collection quality through supervision, and establishing secure IT infrastructure for data management. In addition to the outlined activities for developing a SRS, developing community partnerships, and garnering community buy-in are essential components of the process. Community partnerships can be cultivated through various means, such as establishing dialogue forums, conducting community meetings, and collaborating with community and religious leaders and organisations. Furthermore, involving community members in activities such as mapping clusters, surveys, and interviews will foster a sense of ownership and investment in the data collection process. By actively participating in these activities, community members become stakeholders in the success of the SRSs, thereby increasing their commitment to ensuring accurate and comprehensive data collection.

Building national capacity in terms of human and technical capital will involve coordination among provinces and leveraging partnerships with international collaborators, academia, and the private sector. The Ministry of National Health Services Regulations and Coordination could effectively coordinate activities alongside the Technical Support Unit – CRVS under the Ministry of Planning, Development, and Special Initiatives. Additionally, creating technical training institutes within each province, in collaboration with local academic institutions, can help develop grassroots capacity. Indonesia has proposed a similar strategy for scaling up its current SRS system into a comprehensive CRVS system [32].

The development of an SRS has several strategic benefits and will provide an opportunity to develop and streamline several processes required for CRVS implementation. Furthermore, in addition to providing valuable information to governmental agencies, if UHC is to be implemented in Pakistan, funders, and third-party payers (insurance companies and others) will need to have accurate information on deaths so that they can effectively strategise resources.

#### Strategies to optimise costs of an SRS

The implementation of an SRS, whether on a national or subnational level, requires significant capital investment from internal and external partners. However, if executed strategically by leveraging existing systems, an SRS may prove to be cost-effective in certain contexts and situations. Ultimately, the overriding emphasis of any developed system should be a transition from being funded by external partners to being funded by local stakeholders to ensure long-term effectiveness and sustainability. An example of this transition is the Countrywide Mortality Surveillance for Action project, which was handed over to the Mozambican government after its inception by donor agencies [33]. Pakistan can follow a similar model.

Joshi and colleagues [34] explored the cost-effectiveness of running a VA based mortality surveillance system in India, with costs reported as low as USD 0.10 per capita per year. While costs incurred with SRS implementation include recruiting and training relevant personnel to conduct the VA interviews and establishment of relevant infrastructure (IT and others), they can largely be avoided by integrating the SRS process with already existing healthcare and surveillance infrastructure, such as into national demographic surveillance studies and surveillance infrastructure employed by the Pakistan Bureau of Statistics and NADRA. Another key programme that can be tapped in Pakistan is the Lady Healthcare Worker (LHW) programme, which provide extensive coverage in both rural and urban settings. Moreover, previous research has demonstrated the capability of LHWs to seamlessly integrate technology-based interventions into their operations [35]. Incorporating existing LHWs will integrate community involvement in the process leading to ownership and sustainability. Yet, in recent years, LHWs have been overburdened with involvement in other vertical and horizontal public health programmes, aside from their assigned responsibilities, with limited recruitment since the 90s [36]. In case a future SRS will leverage the knowledge and utility of LHWs on a national scale, if any, it must be done on a judicious basis, with emphasis on fair pay and increased recruitment of LHWs specifically for this purpose.

Significant costs are also incurred with physicians coding the information collected by VA interviews to determine the COD. However, novel open-source algorithms such as InterVA5 and Smart VA Analyze could code VA interviews with reasonable accuracy, exponentially increasing efficiency and saving significant financial resources [37]. Computer-coded algorithms also have the added advantage of standardisation overcoming the challenges of comparability that arise due to differences in physician judgment across populations. Yet, to ensure accuracy, they need to be trained with appropriate and representative large training sets [37]. Furthermore, emerging artificial intelligence algorithms may be able to code the VA interviews and pick up nuances that would otherwise go unrecognised by computer algorithms, hence leading to more accurate COD estimation. Progress in electronic data collection, automated data interpretation, and electronic databases will also foster synergy in terms of sharing data and monitoring and evaluating disease burdens [19]. The learnings from SRS development, particularly regarding data digitisation, data standardisation, and development of data pipelines, can be upscaled to eventual CRVS implementation.

## CONCLUSIONS

Pakistan faces a substantial challenge in addressing the mortality data gap and improving its CRVS system, with multifaceted issues hindering the establishment of an effective mortality surveillance system. The solution proposed here involves a strategic shift towards developing a range of mortality surveillance systems, through which Pakistan could create an agile network of methodologies working in conjunction to provide real-time data on death in a cost-sensitive manner, emphasising cross-sectoral collaboration, capacity building, and local stakeholder involvement. Graded strengthening can allow the country to establish and streamline relevant systems and processes which can then be utilised in a comprehensive CRVS system, which ultimately remains the long-term goal.

## Additional material


Online Supplementary Document
