# Supplementary material for: A systems-change approach to addressing the mortality surveillance gap in Pakistan
Source: J Glob Health. 2025 Aug 4;15:03027. doi: 10.7189/jogh.15.03027 (PMC12319398; doi:10.7189/jogh.15.03027)
Supplement: Online Supplementary Document [file jogh-15-03027-s001.pdf]

Supplement to: Raja MHR, Hoodbhoy Z, Sheikh S, Nisar MI, Soofi SB, Siddiqi S, Mirza Z, Bashir F, Mehmood MT, Samad Z. A systems-change approach to addressing the mortality surveillance gap in Pakistan. J Glob Health. 2025;15:03027.

Figure S1. Multiple cause diagram for a mortality data gap in Pakistan

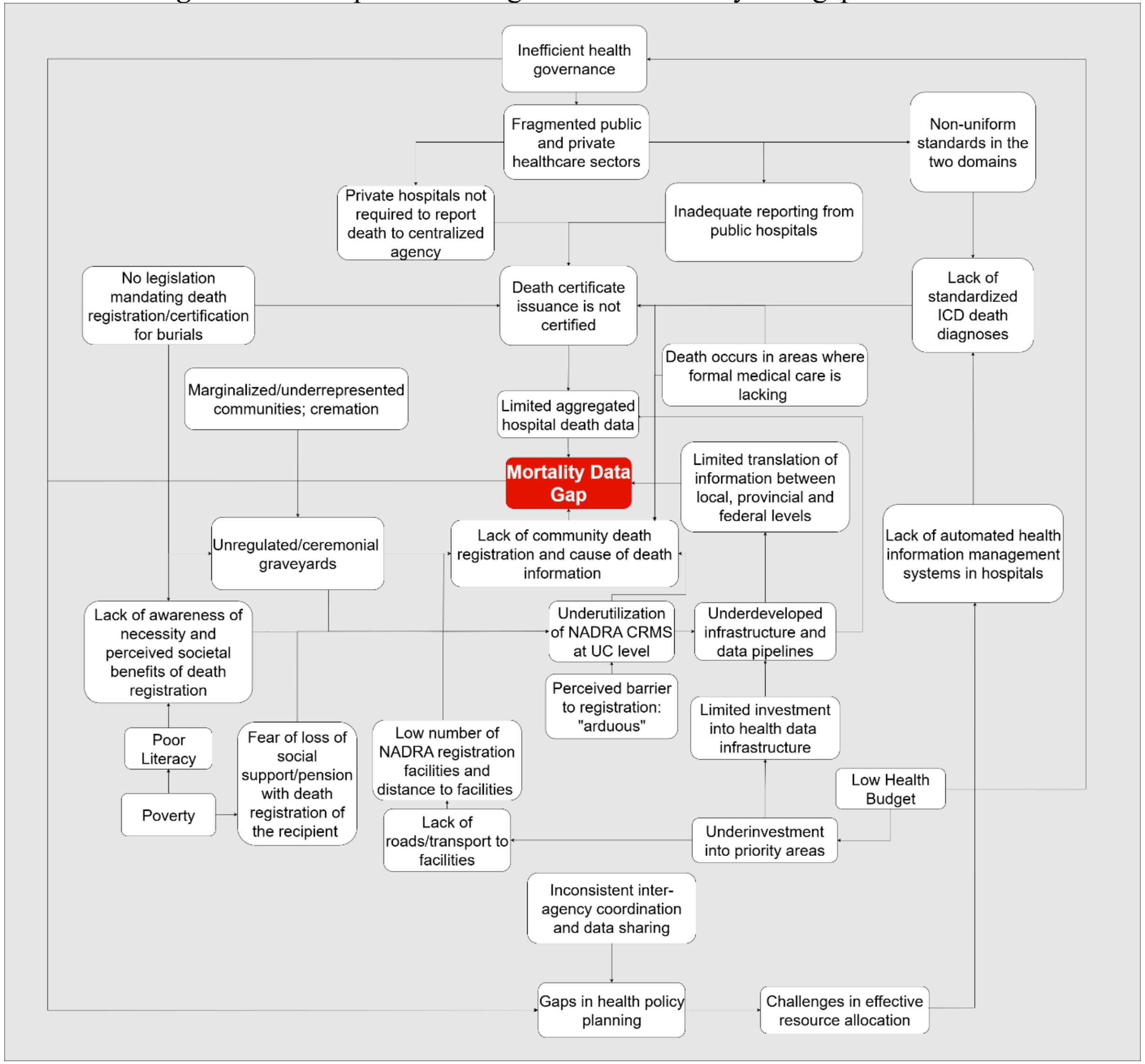

Table S1. List of small-scale community-based or validation studies

Study Title/Year	Type of Study	Location	Population/Sample Size
Cause-specific child mortality in a mountainous community in Pakistan by verbal autopsy/1993 (1)	Cross Sectional	Oshikhandass, Gilgit Baltistan	Children<5 years/52
A community-based nested case-control study of maternal mortality/ 1994 (2)	Case Control	Karachi, Pakistan	Women, 15<x<49/121
Maternal mortality in different Pakistani sites: ratios, clinical causes and determinants/ 1997 (3)	Cross Sectional	Karachi, Sindh<br><br>Lasbela, Pishin, Khuzdar, Loralai, Balochistan<br><br>Abbottabad & Mansehra, Khyber Pakhtunkhwa	Women, 15<x<49/196
Adult mortality in slums of Karachi, Pakistan/ 2000 (4)	Cross Sectional	Karachi, Pakistan	Adult 15<x<59/345
Time to focus child survival programmes on the newborn: assessment of levels and causes of infant mortality in rural Pakistan/2002 (5)	Cross Sectional	Lasbela, Pishin, Khuzdar, Loralai, Balochistan<br><br>Dera Ismail Khan, Hazara, Peshawar Khyber Pakhtunkhwa<br><br>FATA	Neonatal deaths<28 days/649<br><br>Post neonatal deaths 28 days<x<364 days/492

Maternal mortality among Afghan refugees in Pakistan, 1999-2000/ 2002 (6)	Cohort	Hangu, Khyber Pakhtunkhwa	Women, 15<x<49/66
Validation of verbal autopsy to determine the cause of 137 neonatal deaths in Karachi, Pakistan/2003 (7)	Validation	Karachi, Sindh	Neonatal deaths<28 days/137
Demographic and Health Survey 2006-07/2008 (8)	National Survey	Nationwide	Stillbirths/1337<br><br>Children<5 years/3101<br><br>Women, 12<x<49/1062
Validating the verbal autopsy questionnaire for maternal mortality in pakistan/2008 (9)	Validation	Rawalpindi, Islamabad & Hyderabad	Women, 15<x<49/110
Verbal Autopsy of Maternal Deaths in Two Districts of Pakistan—Filling Information Gaps/2009 (10)	Cross Sectional	Sukkur and Malir Districts, Sindh	Women, 15<x<49/128
Maternal mortality in rural community: a challenge for achieving millennium development goal/ 2010 (11)	Cross Sectional	Matiari, Sindh	Maternal Mortality/72
To determine the probable causes of death in an urban slum community of Pakistan among adults 18 years and above by verbal autopsy/2011(12)	Cross Sectional	Nurpur Shahan, Islamabad Capital Territory	Adults>18 years/300

Validation of verbal autopsy tool for ascertaining the causes of stillbirth/2013 (13)	Validation	Karachi & Hyderabad, Sindh	Stillbirths/204
Diagnostic accuracy of WHO verbal autopsy tool for ascertaining causes of neonatal deaths in the urban setting of Pakistan: a hospital-based prospective study/2015 (14)	Validation	Karachi & Hyderabad, Sindh	Neonatal deaths<28 days/626
Using community informants to estimate maternal mortality in a rural district in Pakistan: a feasibility study/ 2015 (15)	Cross Sectional	Chakwal, Punjab	Women, 15<x<49/1424
Trajectory of cause of death among brought dead neonates in tertiary care public facilities of Pakistan: A multicenter study/ 2017 (16)	Descriptive Case Series	Nationwide	Neonatal deaths<28 days/431
Effect of an integrated neonatal care kit on cause-specific neonatal mortality in rural Pakistan/ 2020 (17)	Randomized Control Trial	Rahim Yar Khan (RYK), Punjab	Neonatal deaths<28 days/84

1. Marsh D, Majid N, Rasmussen Z, Mateen K, Khan AA. Cause-specific child mortality in a mountainous community in Pakistan by verbal autopsy. J Pak Med Assoc. 1993;43(11):226-9.
2. Fikree FF, Gray RH, Berendes HW, Karim MS. A community-based nested case-control study of maternal mortality. Int J Gynaecol Obstet. 1994;47(3):247-55.
3. Fikree FF, Midhet F, Sadruddin S, Berendes HW. Maternal mortality in different Pakistani sites: ratios, clinical causes and determinants. Acta Obstet Gynecol Scand. 1997;76(7):637-45.
4. Marsh DR, Kadir MM, Husein K, Luby SP, Siddiqui R, Khalid SB. Adult mortality in slums of Karachi, Pakistan. J Pak Med Assoc. 2000;50(9):300-6.
5. Fikree FF, Azam SI, Berendes HW. Time to focus child survival programmes on the newborn: assessment of levels and causes of infant mortality in rural Pakistan. Bull World Health Organ. 2002;80(4):271-6.
6. Bartlett LA, Jamieson DJ, Kahn T, Sultana M, Wilson HG, Duerr A. Maternal mortality among Afghan refugees in Pakistan, 1999-2000. Lancet. 2002;359(9307):643-9.
7. Marsh DR, Sadruddin S, Fikree FF, Krishnan C, Darmstadt GL. Validation of verbal autopsy to determine the cause of 137 neonatal deaths in Karachi, Pakistan. Paediatr Perinat Epidemiol. 2003;17(2):132-42.
8. Pakistan demographic and Health Survey 2006-07 Islamabad: National Institute of Population Studies; 2008. .
9. Midhet F. Validating the verbal autopsy questionnaire for maternal mortality in pakistan. Int J Health Sci (Qassim). 2008;2(1):91-6.
10. Jafarey SN, Rizvi T, Koblinsky M, Kureshy N. Verbal autopsy of maternal deaths in two districts of Pakistan--filling information gaps. J Health Popul Nutr. 2009;27(2):170-83.
11. Nisar N, Sohoo NA. Maternal mortality in rural community: a challenge for achieving millennium development goal. J Pak Med Assoc. 2010;60(1):20-4.
12. Abbas SM, Alam AY, Majid A. To determine the probable causes of death in an urban slum community of Pakistan among adults 18 years and above by verbal autopsy. J Pak Med Assoc. 2011;61(3):235-8.
13. Nausheen S, Soofi SB, Sadiq K, Habib A, Turab A, Memon Z, et al. Validation of verbal autopsy tool for ascertaining the causes of stillbirth. PLoS One. 2013;8(10):e76933.
14. Soofi SB, Ariff S, Khan U, Turab A, Khan GN, Habib A, et al. Diagnostic accuracy of WHO verbal autopsy tool for ascertaining causes of neonatal deaths in the urban setting of Pakistan: a hospital-based prospective study. BMC Pediatr. 2015;15:144.
15. Mir AM, Shaikh MS, Qomariyah SN, Rashida G, Khan M, Masood I. Using community informants to estimate maternal mortality in a rural district in Pakistan: a feasibility study. J Pregnancy. 2015;2015:267923.
16. Mustufa MA, Sheikh MA, Taseer IU, Raza SJ, Arshad MS, Akhter T, et al. Trajectory of cause of death among brought dead neonates in tertiary care public facilities of Pakistan: A multicenter study. World J Pediatr. 2017;13(1):57-62.
17. Duby J, Pell LG, Ariff S, Khan A, Bhutta A, Farrar DS, et al. Effect of an integrated neonatal care kit on cause-specific neonatal mortality in rural Pakistan. Glob Health Action. 2020;13(1):1802952.
